# A guide for the diagnosis of rare and undiagnosed disease: beyond the exome

**DOI:** 10.1186/s13073-022-01026-w

**Published:** 2022-02-28

**Authors:** Shruti Marwaha, Joshua W. Knowles, Euan A. Ashley

**Affiliations:** 1 Department of Medicine, Division of Cardiovascular Medicine, School of Medicine, Stanford University, Stanford, CA USA; 2 Stanford Center for Undiagnosed Diseases, Stanford University, Stanford, CA USA; 3 Department of Medicine, Diabetes Research Center, Cardiovascular Institute and Prevention Research Center, Stanford, CA USA; 4 Department of Genetics, School of Medicine, Stanford University, Stanford, CA USA

**Keywords:** Diagnosis, Rare, Omics, Exome-negative, Long read

## Abstract

Rare diseases affect 30 million people in the USA and more than 300–400 million worldwide, often causing chronic illness, disability, and premature death. Traditional diagnostic techniques rely heavily on heuristic approaches, coupling clinical experience from prior rare disease presentations with the medical literature. A large number of rare disease patients remain undiagnosed for years and many even die without an accurate diagnosis. In recent years, gene panels, microarrays, and exome sequencing have helped to identify the molecular cause of such rare and undiagnosed diseases. These technologies have allowed diagnoses for a sizable proportion (25–35%) of undiagnosed patients, often with actionable findings. However, a large proportion of these patients remain undiagnosed. In this review, we focus on technologies that can be adopted if exome sequencing is unrevealing. We discuss the benefits of sequencing the whole genome and the additional benefit that may be offered by long-read technology, pan-genome reference, transcriptomics, metabolomics, proteomics, and methyl profiling. We highlight computational methods to help identify regionally distant patients with similar phenotypes or similar genetic mutations. Finally, we describe approaches to automate and accelerate genomic analysis. The strategies discussed here are intended to serve as a guide for clinicians and researchers in the next steps when encountering patients with non-diagnostic exomes.

## Background

Although the occurrence of individual rare diseases often seems negligible, it is estimated that 30 million people in the USA are suffering from a rare disease, affecting 1 in 10 Americans, equivalent to the prevalence of type 2 diabetes [1, 2]. About 7000 rare disorders are defined [2, 3] and many others fall under the umbrella of undiagnosed diseases. Most patients suffering from a rare or undiagnosed disease receive only symptomatic treatment. An accurate diagnosis can result in better management of the disease, identification of potential therapeutics and avoid unnecessary treatments that may have severe side effects. For inherited rare diseases, knowing the causative variant and the mode of inheritance informs patients about the risk of passing the disease to future generations and helps evaluate alternate family planning options [4]. The diagnostic delay for rare diseases varies from months to decades, depending on the patient’s phenotype, age, and available resources. The average time for accurate diagnosis of a rare disease is about 4–5 years [5–7]; in some cases, it can take over a decade [8, 9]. These patients face a diagnostic odyssey and often undergo extensive and expensive workups at several institutions. Despite this, patients often remain undiagnosed or even misdiagnosed [8], which further adds emotional distress to patients and family members.

**Fig. 1:**
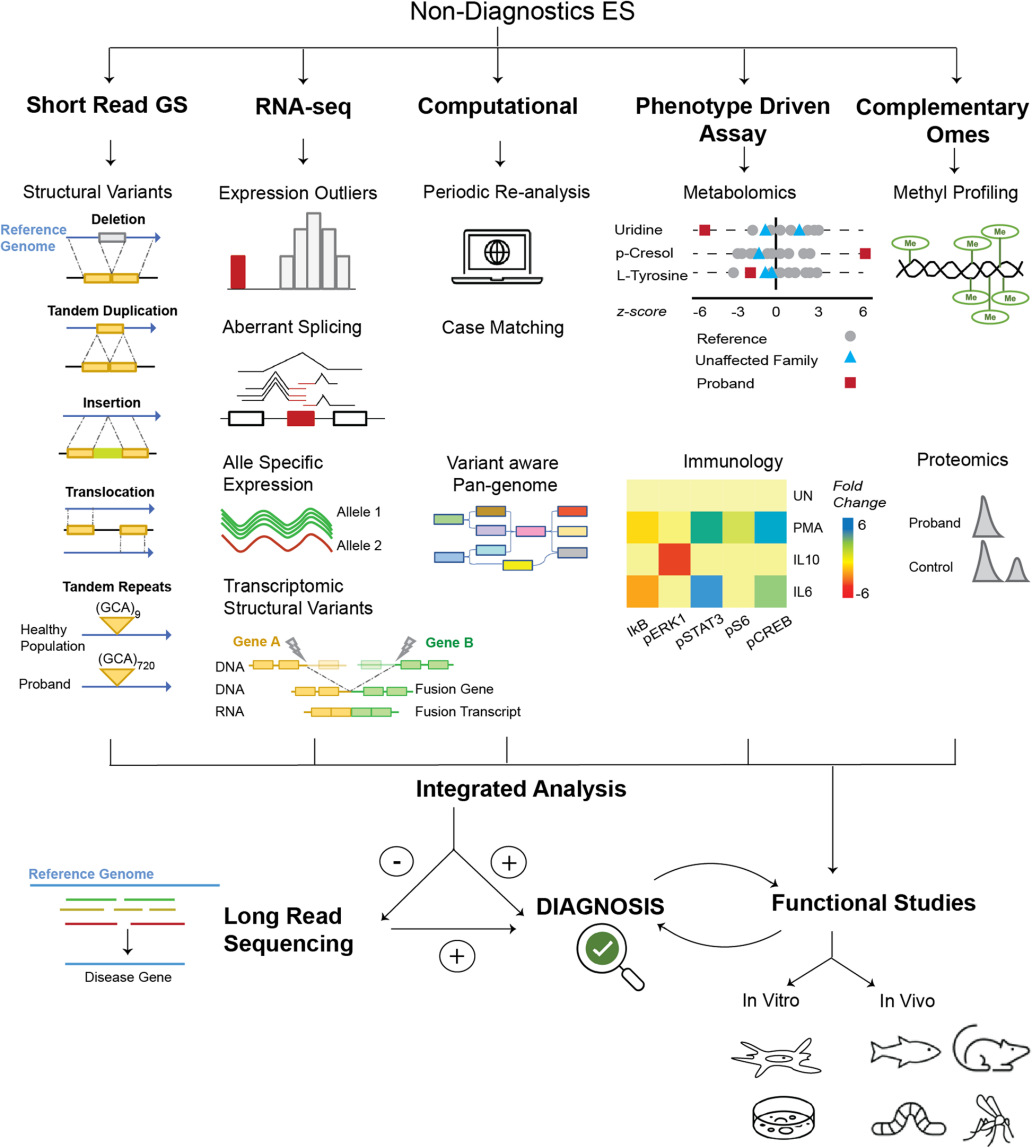
Technologies and methods to diagnose rare diseases when ES is unrevealing. Many of these technologies complement each other, fill the missing gaps, and should be analyzed in an integrated manner. The selection between metabolomics, proteomics, methyl profiling, and immune assays should be guided by the patient’s clinical presentation and existing candidate genes identified through sequencing. Functional studies can be used to validate strong candidate variants or elucidate the underlying molecular mechanism of the disease after identifying the causative gene. ES: Exome Sequencing, GS: Genome Sequencing, UN: unstimulated. Some of the graphics representing different technologies or methods have been adapted with permission from [10–12]

**Fig. 2:**
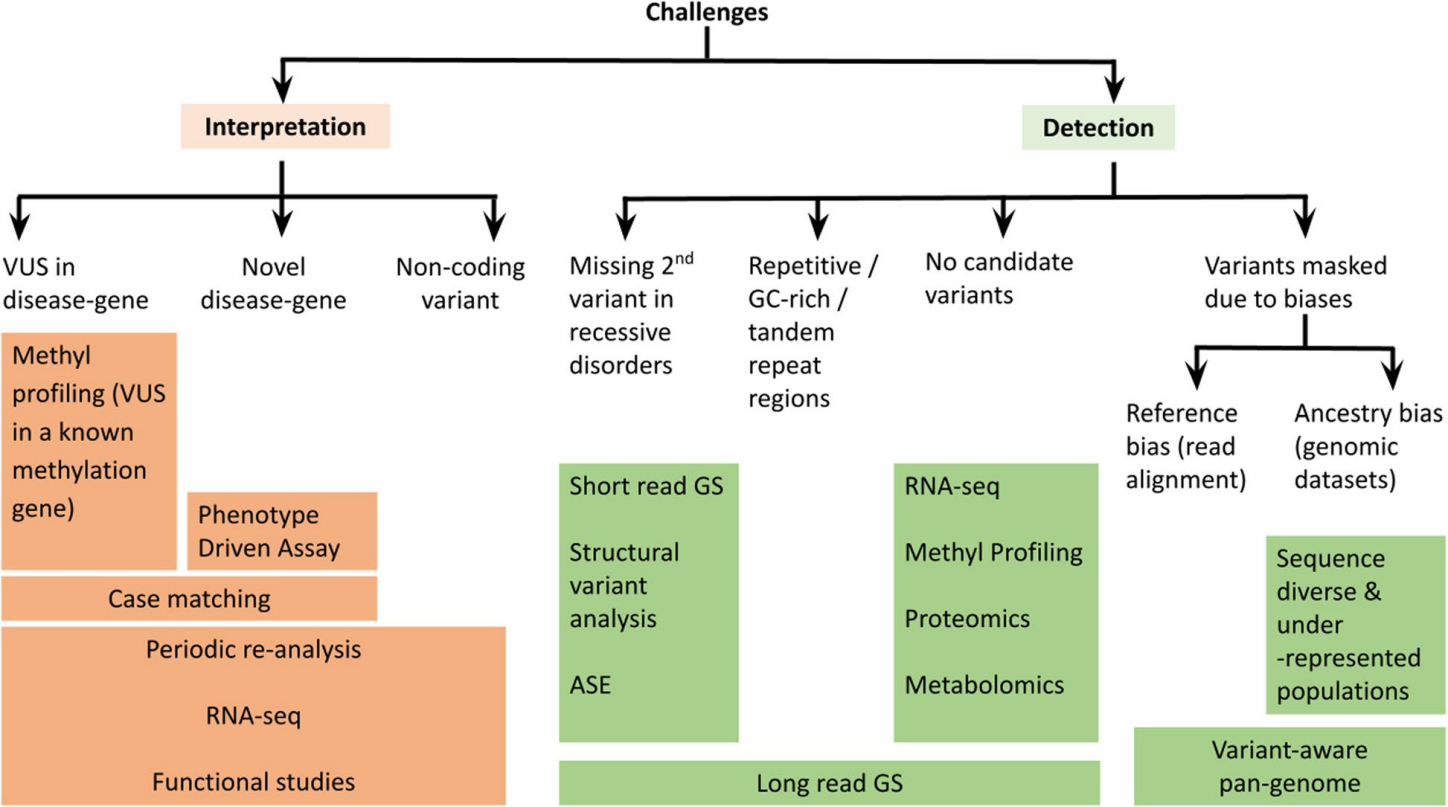
Challenges in identifying causal variants using exome or genome sequencing and the potential solutions and alternate approaches. These challenges can be at the level of interpretation or detection. GS: genome sequencing, ASE: allele-specific expression, VUS: variant of unknown significance

It is estimated that 80% of rare diseases have a genetic origin [13]. Until 10 years ago, genetic testing was expensive and usually limited to a few genes at a time. The advent of next-generation sequencing technology has had a dramatic effect on the cost, accuracy, and utility of genetic testing and has supplanted older technologies. Many undiagnosed diseases [14, 15] have been identified by exome sequencing (ES) that looks at the protein-coding regions, which constitute less than 2% of the genome [16]. Additionally, sequencing family members and performing segregation analysis can eliminate hundreds of non-causative variants and thus reduce the search space. In a cohort of children with undiagnosed developmental disorders (*n* = 989) and unaffected parents, Wright et al. [17] observed that exome sequencing of the parent–child trios rather than singletons reduced candidate variants by ten folds. Clark et al. [18] performed a meta-analysis on five studies consisting of children with suspected genetic diseases (*n* = 3613) to compare the diagnostic yield of genome sequencing (GS)/ES by individual proband and trio testing within cohorts. They found that the odds of diagnosis using trios was double that using singletons.

Programs like Care for Rare [19], Deciphering Developmental Disorders [20], Rare and Undiagnosed Diseases Diagnostic Service [21], and the Undiagnosed Diseases Network [22] have demonstrated how exome sequencing can not only end an expensive, potentially invasive and emotionally challenging journey for the patients but also help in better disease management [23, 24]. Still, a minority of patients receive a definitive molecular diagnosis [17, 25–28]. This review, aimed towards clinicians and rare disease researchers, presents the key challenges in diagnosing patients with negative exome sequencing and discusses the strategies that can potentially fill the diagnostic gap in such patients. We propose technologies that should be considered when ES is unrevealing, many of which complement each other (Fig. 1). These include sequencing the whole genome and the transcriptome. Based on the patient’s phenotype, metabolomics, or proteomics or methyl profiling should be considered. In parallel, automated processes should be established for periodic re-analysis of the genomic data and identification of patients with similar phenotypes or similar genetic mutations. Finally, functional studies should be conducted to support the causality of a putative variant and understand the molecular mechanism of the rare disease.

## Genome sequencing

ES can capture the protein-coding regions of the genome and in some cases also untranslated regions (UTRs) and intron-exon boundaries [29], at a low cost. In addition, augmented exome capture techniques can further improve the coverage in medically relevant genes [30]. Despite the numerous advancements of exome sequencing, it has non-uniform coverage (particularly in first exons, regions of high GC/AT, and regions of low complexity [31–33]) and is limited by the specificity of the capture probes [34]. ES has had modest success in detecting structural variants, tandem repeats, and pathogenic variants in deep intronic regions. Some of these challenges can be addressed by genome sequencing (GS) [31, 33, 35, 36]. GS can identify canonical [37, 38] and complex structural variants [39, 40], tandem repeats [37, 38], intronic variants [37, 38], and coding variants that may not be accurately captured by ES. GS has enabled identification of the causative variants for many undiagnosed cases where prior ES was either unrevealing [38, 41] or had provided only partial diagnosis (the causative variant explained only some phenotypes of the patient) [42]. Diagnosis mediated by GS has also opened avenues for therapy in some cases by identifying the disease mechanism and potential drug targets [42–44]. In this section, we illustrate with examples how short-read genome sequencing technology can facilitate detection of structural variants and tandem repeats that are often missed by ES. We discuss different long-read sequencing platforms, its advantages over short-read, especially for detecting large, complex structural variants and methylation changes and explore the potential of pan-genome reference in aiding rare disease diagnostics.

### Structural variants

Structural variants (SVs) represent a class of variants that are greater than 50 base pairs (bp) [10, 45–47] and can be as long as 3 Mb [48, 49]. Structural variants include microscopic and often submicroscopic variants that comprise deletions, duplications, insertions, inversions, mobile element insertions (transposons), translocations, and complex rearrangements [10]. Since SVs often encompass several exons or genes, GS is a better tool for studying them than ES. With the advent of PCR-free library preparations, population frequency databases [47, 50, 51], benchmarking structural variant datasets [52], and recent advancements in SV detection algorithms [46, 53], many groups have implemented GS to identify pathogenic SVs in previously undiagnosed patients [39, 54–57]. In a cohort of 477 undiagnosed patients with varied phenotype, Holt et al. [55] identified molecular diagnoses for 16 cases (3.35%) by scanning for structural variants using short-read GS data. Carss et al. [57] showed that in a phenotypically heterogeneous group of 722 inherited retinal disease (IRD) patients, 33 pathogenic structural variants were responsible for the disease in 31 (4.29%) individuals. Despite the ability of GS to detect large and complex variants, their interpretation remains difficult, especially for the non-coding variants [58]. The challenges associated with identifying the causative variant from ES/GS can be broadly classified into two groups — (i) interpretation (VUS in a known disease gene, novel disease gene or non-coding variant) and (ii) detection (missing second variant in a recessive disorder, causative variant lies in difficult to sequence region or is masked due to biases in reference genome and genomic datasets). In Fig. 2, we summarize these challenges and suggest alternate technologies, informatics tools, and experimental approaches that may help to reach a molecular diagnosis. With active development in variant prioritization algorithms (like genomiser [59], SpliceAI [60]), new disease-gene discoveries, and complementary omic technologies (like RNA-seq, metabolomics, and proteomics), we expect the diagnostic yield by GS will continue to improve.

Short tandem repeats (STRs) are short (1–6 bp) DNA sequences repeated head-to-tail multiple times. Approximately 3% of the human genome consists of STRs [61] and 6% of human coding regions are estimated to contain STR variation [62]. Expansion of non-coding repeats can result in loss of protein function or altered RNA function while expansion of coding repeats can cause altered protein function [63]. STRs have been implicated in many neurological and genetic diseases like Friedreich’s ataxia (GAA), Huntington’s disease (CAG), Fragile X Syndrome (CGG), amyotrophic lateral sclerosis (GGGGCC), and other hereditary ataxias [63–68]. Historically, STRs were genotyped using polymerase chain reaction (PCR) and gel electrophoresis, which is time consuming, costly and limited to identifying expanded repeats in regions previously associated with STR diseases. In past years, many bioinformatics tools like GangSTR [69], ExpansionHunter Denovo [70, 71], and STRetch [72] have been developed to predict STRs from PCR-free short-read sequencing. A recent study [73] benchmarked 8 STR prediction tools using known disease-causing full-mutation STR expansions and simulated data and found that the ensemble approach of using ExpansionHunter, STRetch, and exSTRa performed the best. These tools have enabled diagnoses of many Mendelian diseases caused by repeat expansion [74–76]. We discuss one of the cases in detail to demonstrate how STR analysis can guide diagnosis when ES is inconclusive.

Using GS and clinical and biochemical phenotyping, Kuilenburg et al. [75] identified expansion of GCA-repeat region in 5′UTR of glutaminase gene (*GLS*) in three unrelated patients with an inborn error of metabolism that resulted in reduced glutaminase activity. Initially, exome sequencing of the three patients and their families identified heterozygous, damaging variants in *GLS* gene in probands 1 and 3. Although a good phenotypic match, the ES finding was not conclusive and prompted further biochemical analysis and genome sequencing of proband 1. Expansion Hunter tool that identifies repeat expansions in a locus-specific manner, predicted a large GCA-repeat expansion in proband 1 when compared with control population (*n* = 8295). Later, large GCA-repeat expansions in the *GLS* were confirmed in all three patients using triplet repeat-primed PCR assay, facilitating molecular diagnoses for the three patients.

Although short-read sequencing can potentially identify known and novel SVs and repeat expansions across the genome, it has limited success in detecting large, complex structural variants and long tandem repeats or those which lie in highly repetitive and/ or GC-rich regions [77]. In the following section, we address how some of these limitations can be overcome by long-read sequencing.

### Long-read sequencing

Reads from short-read sequencing (SRS) — typically 100–300 bp long [78]— are mapped to a consensus reference during the alignment step. Nowadays, paired-end sequencing (sequencing both ends of a fragment) is often performed over single-end, allowing more accurate mapping of the reads, especially in regions with repetitive sequences. However, the alignment process is still challenging in repetitive regions of the reference genome because of the short length of reads, making it difficult to predict large variants and long tandem repeats with high certainty. In the last decade, new sequencing technologies have been developed that generate reads that typically range 10–60 kb [79], with some extending to 2 Mb [80]. The longer reads result in improved alignment to the reference genome and better detection of SVs, especially within repetitive elements or segmental duplications or high GC content, regions that were difficult to access using short-read technology [81].

Long-read sequencing (LRS) also allows haplotype phasing — assigning genetic variants to the homologous paternal or maternal chromosomes [82, 83]. This information helps in identifying compound heterozygous mutations and de novo autosomal dominant mutations [84]. Most variant callers developed for short-read sequencing provide unphased variants and thus require sequencing of parents to detect compound heterozygous and de novo mutations. LRS also provides precise details about the breakpoints [85], improving our understanding of the mutation and disease mechanism.

Currently, there are two main LRS platforms — single-molecule real-time (SMRT) sequencing from Pacific Biosciences [86] and nanopore-based sequencing from Oxford Nanopore Technologies (ONT) [87]. ONT can generate very long contiguous reads (2.2 Mb) [80] while Circular Consensus Sequencing (by Pacific Biosciences) provides highly accurate (99.8%) high-fidelity (HiFi) reads with an average length of 13.5 kb [88]. Cost-effective synthetic LRS technologies like linked reads (Transposase Enzyme Linked Long-read Sequencing (TELL-seq) [89], single-tube long fragment read (stLFR) [90], Hi-C and chromatin cross-linking [91, 92], and optical mapping (from BioNano Genomics) [93] can provide several advantages of LRS (e.g., detection of large SV) at some additional cost. We suggest the review by Sedlazeck et al. [81] and Sakamoto et al. [94] for further in-depth comparison of these technologies.

LRS also enables direct detection of methylated nucleotides [95–97]. Among all types of methylation modifications in DNA, 5-methylcytosine (5mC) is most well studied, partly due to the advancements in bisulfite-based short-read sequencing techniques like whole genome bisulfite sequencing (WGBS) [98] and reduced representation bisulfite sequencing (RRBS) [99, 100]. Although bisulfite sequencing provides a quantitative and accurate measure of 5mC modifications at base resolution, it cannot capture other methylation changes including 6-methyladenine (6 mA) and 4-methylcytosine (4mC). In contrast, long-read technology sequences native DNA and can predict the base modifications from the deviations seen in the raw signal, thus avoiding DNA amplification and bisulfite conversion steps and the biases associated with them [101]. Both nanopore technology and SMRT sequencing can capture many types of base modifications (including 5mC, 4mC, and 6 mA) simultaneously. In Pacific Biosciences’ SMRT sequencing, DNA polymerase adds labeled nucleotides along the template DNA, generating a succession of fluorescence pulses. Base modification can alter the kinetics of the polymerase during this process. If a nucleotide is methylated, DNA polymerase will pause before incorporating the next nucleotide. Changes in fluorescence pulses from the labeled nucleotides are used to measure the shift in polymerization speed, thus detecting the base modification. For example, the time interval between two successive fluorescence pulses called inter-pulse duration is used to detect 6 mA [81, 95]. Recently, Tse OYO et al. [102] developed a method that drastically improved the detection of 5mC modifications from SMRT sequencing using sequence context, inter-pulse duration, and pulse width associated with DNA polymerase kinetics. ONT’s technology identifies one of the 5 possible nucleotides based on the difference in electrical current produced when the base passes through protein nanopores embedded in a flow cell. Base modifications on the DNA or RNA cause a minor shift in current that can be detected and interpreted by algorithms [96, 103]. Comparison of performance of nanopore, SMRT, and bisulfite-based short-read sequencing on the same set of samples will inform the community about benefits and limitations of each technology and which method is most suitable under a given situation.

Using low coverage LRS, Merker et al. [56] identified a 2184-bp deletion in a patient with negative targeted clinical testing and unrevealing SRS of the genome. Maio et al. [4] demonstrated how LRS can help to solve recessive disease cases where the second pathogenic allele is missing from the ES data. Although SR GS is capable of identifying repeat expansions, it is limited in detecting undiscovered repeat diseases in long [104] and complex GC-rich regions [105]. Recent studies have proved that LRS can detect known [106, 107] and novel repeat expansions [104, 108, 109] for Mendelian diseases in which no causal variants were detected through SRS. The longer reads can encompass an entire expanded repeat or a flanking unique sequence, making long-read technology apt for analyzing tandem repeat expansions [104]. Facioscapulohumeral muscular dystrophy 1 (FSHD1) disease results from a heterozygous contraction of 3.3 kb repeat unit (referred as D4Z4) in the subtelomeric region of chromosome 4q35 and a chromosome 4 haplotype called 4qA. The D4Z4 unit varies from 11 to 100 repeats in the healthy population, but FSHD1 patients show only 1–10 repeats, hence the term contraction [110]. Conventionally Southern Blotting is used for molecular diagnosis of FSHD1 and alternative methods have been explored as Southern Blot is semi-quantitative and time consuming. However, sequencing long, repetitive subtelomeric regions of the genome is challenging for both short-read technology and Sanger sequencing. Moreover, D4Z4 has variable number of repeat units and homologous repeat array on chromosome 4 and chromosome 10. Both true LR (SMRT [111] and ONT’s Minion [112]) and synthetic LR (BioNano Genomics’ optical mapping) [113] technologies have shown variable degrees of success in sequencing this region, determining the repeat number and haplotype. With continuing improvements, long-read technologies can enable sequencing of more such difficult-to-sequence regions, identifying new associations between genomic regions and genetic disorders. Several groups have exemplified the clinical significance of targeted long-read sequencing using CRISPR/Cas9 [114] mediated methods [115, 116] or computational adaptive sampling [117] for enrichment of specific regions of the genome. In a small cohort of 22 patients with known canonical and complex SVs, Miller et al. [117] demonstrated that targeted LRS can not only detect all SVs previously identified with clinical testing (*n* = 46) but also discovered variants (*n* = 41) that were missed by the clinical test. Targeted long-read sequencing has the potential to be used clinically for patients with suspected complex SVs and tandem repeats in candidate genes.

In summary, LRS can be the single test to detect single-nucleotide variants (SNVs), insertion and deletion (INDELs), simple and complex SVs, tandem repeats, and methylation changes and inform about phasing. Although LRS holds promise for undiagnosed genetic diseases, some challenges need to be overcome in order to bring LRS from the research setting to the clinic. The cost of ONT is now comparable to SR but Pacific Biosciences is relatively expensive. Recently, great strides have been made in sequencing technology and algorithm development to improve the accuracy of calling small variants (SNVs and INDELs) from nanopore and HiFi long-read data [88, 118]. At high coverage, both long-read sequencing platforms can outperform the short-read-based method in accurate SNV identification at whole genome scale, including segmental duplication and difficult-to-map regions. SR and HiFi have comparable performance at identifying INDELs but ONT has much lower accuracy. Identification of base modifications (epigenetic changes) by LRS is still in its infancy and suffers from low accuracy and the need for training models [101]. However, continuous improvements in sequencing technology and algorithm development are being made to further increase the accuracy and lower the cost, which will eventually enable the use of LRS routinely for clinical diagnostics. In the interim, an attractive solution is a hybrid approach combining the advantages of each in a combined assay using, for example, lower coverage (e.g., 15×) long-read sequencing along with higher coverage (e.g., 40×) short-read sequencing or using targeted LRS to evaluate candidate genes or in case of suspected tandem repeat disease or complex rearrangements.

### Pan-genome reference

The current human reference genome is a linear haploid consensus sequence derived from a very small number of individuals and thus lacks genetic diversity observed across populations [119, 120]. Mapping sequencing reads to this reference genome can cause the reads to be misaligned or remain unaligned, especially in highly polymorphic or repetitive regions or regions spanning structural variant breakpoints [119, 121] or may miss a rare variant that is represented by the minor allele on the haploid reference sequence [122]. This results in a “reference bias” as non-reference alleles from a sample are difficult to align to the linear reference sequence. To overcome these limitations, many efforts have been made in the past few years to incorporate known variants in the reference in order to allow variant-aware read alignment and variant calling [119, 120, 123–125]. These efforts propose a pan-genome, which represents a collection of all genomic sequences in a population or a species or a phylogenetic clade [123, 126].

Aligning reads to a pan-genome that considers many alternate haplotypes at each locus reduces the reference bias [127], thereby improving alignment accuracy and variant calling [119, 124]. Recently, Siren et al. [120] developed a tool called Giraffe to map short reads to pan-genome with high accuracy and speed. Giraffe detected SNVs, INDELs, and SVs more accurately when using a pan-genome than using the single reference genome, showcasing the significance and practicality of the pan-genomic approach to short-read mapping. It was able to genotype 167,000 SVs that were discovered from LR studies, in 5202 individuals from diverse populations that were sequenced by SR sequencing. Recently, precisionFDA truth challenge V2 evaluated different bioinformatics pipelines’ accuracy in predicting small variants in difficult-to-map regions and Major Histocompatibility Complex using Genome In A Bottle (GIAB) benchmark data set [128]. The top performing algorithms in the short-read sequencing category used either alt-aware mapping (DRAGEN’s graph mapper) or pan-genome (by Seven Bridges’ GRAF pipeline). Despite the several benefits of the pan-genome, there are practical limitations associated that have hindered the community in embracing this paradigm shift. This includes high compute cost, scalability, and complexity of the tasks. Addition of variants to the existing linear reference genome is not straightforward as simply adding more variation to the reference can result in more ambiguity. Alternative methods have been proposed that aim to strike a balance between accuracy and limitations of graph-based pan-genome. Reference flow [129] involves an iterative two-step process. Reads are first aligned to the linear reference genome and the unaligned reads and the reads with low mapping-quality are then re-aligned to a set of references. Tetikol et al. [130] recommend population-specific graphs that iteratively augment tailored genome graphs for targeted populations.

By reducing the reference bias, the graph genome will be instrumental in detecting novel structural variants, large INDELs, and mutations that affect allele-specific expression [126, 131, 132]. The pan-genomic model can help to detect more accurate variants for rare disease patients from underrepresented populations [130] and even allow construction of personalized reference genome using the parent’s sequencing data (if available). To the best of our knowledge, pan-genome has not yet led to a diagnosis; however, efforts are being made in this direction [133]. In the next few years, we expect further optimizations in speed and accuracy of the tools working in the pan-genome space. Since use of pan-genome and variant-aware algorithms lead to more accurate variant detection in SR sequencing, especially the structural variants, we anticipate that these approaches will benefit diagnoses of ultra-rare patients.

## Transcriptomics

Although genome sequencing can theoretically capture all types of variants, prioritization and interpretation of the non-coding variants remains a big challenge. Complementing DNA sequencing with transcriptomics can help to prioritize potential disease-causing variants. In this section, we review four approaches to analyze RNA sequencing data for prioritizing candidate genes for rare diseases — expression outliers, aberrant splicing, allele-specific expression, and transcriptomic structural variants [11, 134–136]. Next, we discuss the potential of long-read sequencing to predict alternative splicing and gene fusions with high accuracy. We also highlight the potential of single-cell transcriptomics to elucidate the cellular and molecular mechanisms of rare and undiagnosed diseases that involve rare, undiscovered cell populations.

RNA sequencing can help to classify a variant of unknown significance (VUS) and provide insights into the disease mechanism or identify variant in the second allele in a recessive disease where genomic sequencing returned only one pathogenic variant [137]. In a cohort of 50 patients with rare muscle disorders, who had non-diagnostic ES and/GS, Cummings et al. [138] illustrated the utility of RNA sequencing the affected tissue (muscle), yielding a diagnostic rate of 34%.

Gene expression and mRNA isoforms can vary significantly from one tissue to another [139, 140] and so it is recommended to use the affected tissue for RNA sequencing [138, 141]. But the disease-relevant tissue is not always easily available in a non-invasive manner. Blood, fibroblasts, and induced pluripotent stem cells (iPSCs) appear to be promising alternatives [11, 134, 141, 142]. By RNA sequencing blood from 94 undiagnosed patients, representing 16 distinct disease categories, Fresard et al. [134] identified the causative variants in 7.5% of the cases, demonstrating the potential of blood transcriptome sequencing to aid the diagnoses of rare Mendelian diseases. Lee et al. [142] reported a 14.5% (*n* = 7) diagnostic rate by sequencing mRNA from blood, fibroblast, and/or muscle samples from 48 genome-negative individuals, primarily affected by neurological (*n* = 25) and musculoskeletal disorders (*n* = 12). They identified pathogenic splicing abnormalities in seven patients with neurological or musculoskeletal diseases. They observed that fibroblast was a better tissue choice than blood for identifying the splicing defects in this cohort. Similarly, Baynam et al. reported a case of megalencephaly-capillary malformation syndrome where the causative mutation (mosaicism in PIK3CA) was detected in fibroblasts and not in blood [21].

However, many genes are expressed at very low levels in both blood and fibroblasts to be captured at high depth by RNA sequencing. CRISPR/Cas9 technology can be used to improve coverage of low-expressed genes in a scalable manner [143]. Huang et al. [144] applied CRISPRclean method, using Cas9 nuclease and 360,000 guide RNAs to specifically remove RNA-Seq library fragments from over 4000 targeted genes and observed about a sixfold increase in coverage of untargeted genes compared to untreated RNA-Seq libraries. iPSCs are a good substitute when the candidate gene is known to be expressed at low levels in blood and fibroblast. Recently, Bonder et al. [145] unified data from five major iPSC genetic studies [146–150] to create the integrated iPSC QTL (i2QTL) consortium. They observed a fivefold enrichment of outliers in known rare disease genes as compared to non-disease genes and demonstrated detection of gene outliers in patients with Bardet-Biedl syndrome and hereditary cerebellar ataxia. Therefore, alternate tissues like fibroblasts, iPSCs, and blood should be considered carefully when the affected tissue is not available for transcriptome analysis. In the following section, we will discuss how sequencing the transcriptome can uncover pathogenic mutations, missed by studying genomic variants alone.

### Expression outliers

When working with rare and undiagnosed diseases, it is assumed that most of the samples express each gene within its physiological range and the goal is to identify genes from each sample that are expressed at extremely high or low levels. This is achieved by calculating *Z*-scores, comparing each patient against others in the cohort. GTEX [140] and GEUVADIS [151] are great resources for additional control RNA-seq samples.

Caution should be exercised when applying the expression outlier approach. For example, controls should be from same tissue type as the disease samples [135]; data should be normalized for batch effect, sex, or biopsy site [11]. Typically, the *Z*-score-based approach uses an arbitrary threshold for selecting outlier genes [134, 138, 141] often followed by applying additional filters like predicted pathogenicity, minor allele frequency, and phenotypic match to further prune down the number of candidate genes [134]. Recent methods like OUTRIDER [152] and PEER [153] control for technical and biological variations among genes and the former also provides a statistical test for outlier detection in RNA-seq samples. Fresard et al. [134] demonstrated how their expression outlier pipeline prioritized a causative gene (*MECR*) within the top 15 candidate genes for two siblings with MEPAN disease. Overall, analyzing expression outliers along with genomic variants and the patient’s phenotype can be a powerful strategy to identify strong candidate variants for clinical interpretation.

### Aberrant splicing variants

Alternative splicing is a naturally occurring phenomenon in eukaryotes that results in a single gene coding for multiple proteins. Post transcription, non-coding sequences (introns) are removed from the pre-mRNA and some exons may be included or excluded from the final, processed mRNA [154]. Errors in this process cause several diseases including rare Mendelian diseases [155]. Splicing mutations can be broadly divided into five categories: exon skipping, inclusion of intronic pseudoexon, exon extension, exon retraction, and intron retention [142, 156]. Algorithms like LeafCutterMD [157] and Fraser [158] provide statistical frameworks that are designed for predicting splicing outliers in rare diseases.

Certain types of variants like synonymous and deep intronic variants are often filtered out by prioritization pipelines unless they have been previously associated with a disease. Such variants can lead to aberrant splicing events and it is possible to re-prioritize them using transcriptomic data [134, 158]. Lee et al. [142] have shown how RNA sequencing helped to identify the second variant in a 2-year-old girl who had an inconclusive trio ES, which reported a paternally inherited frameshift mutation in *SEPSECS* gene (OMIM 613009), associated with autosomal recessive pontocerebellar hypoplasia, type 2d [159]. GS did not reveal any pathogenic maternally inherited coding variant. However, transcriptomics data from the proband and the mother showed that half of their reads in the *SEPSECS* gene skipped exon 7, which carried a synonymous variant. This was missed earlier because typically, synonymous variants are filtered during variant prioritization of genomic data unless they are previously reported to be pathogenic.

### Allele-specific expression

Allele-specific expression (ASE) is a phenomenon in diploid or polyploid genomes, where one allele has significantly higher expression than the other allele [160, 161]. When prioritizing variants from ES/GS data using recessive mode of inheritance, single heterozygous rare variants are filtered out. However, some of these heterozygous rare variants may exhibit ASE. Gonorazky et al. [141] reported that the allele imbalance approach provided diagnostic leads in three monogenic neuromuscular disorder patients, who previously had non-diagnostic ES and/or gene panel results. Kremer et al. [11] discuss how their ASE pipeline helped to establish the genetic diagnosis in a patient with mucolipidosis, who had tested negative for the enzymatic tests available for mucolipidosis type 1, 2, and 3 in blood leukocytes. They detected borderline non-significant low expression in an intronic variant in *MCOLN1* gene that was filtered by their ES pipeline as it was intronic. Therefore, along with identifying expression outliers and splicing variants, ASE analysis should be performed as part of regular RNA-seq analysis, especially when genomic data identifies only one heterozygous variant for a recessive disorder.

### Transcriptomic structural variants

Structural variants (SVs) like translocations, duplications, inversions, and deletions join different genomic regions together or separate one region into pieces. Transcription of such regions can result in gene fusions (exons from two or more distinct genes are transcribed together) or cause a previously non-transcribed region to be included into a gene, often leading to altered gene function in both the cases. Such modifications in the transcribed mRNA that are caused by genomic SVs are known as transcriptomic structural variants (TSVs) [162, 163].

Fusion genes are well documented in hematological and solid tissue cancers and are used as biomarkers for early diagnosis and therapeutic targets [164]. Independent case studies have reported fusion transcripts in many non-cancer diseases like brain malformation [165, 166], intellectual disability [167, 168], spastic paraplegia [169], and Gille de la Tourette Syndrome [170]. Oliver et al. [136] tailored a fusion identification pipeline for rare disease patients and applied it to a cohort of 47 individuals who previously had negative or partial diagnoses through exome sequencing. They identified eight fusion events that were confirmed using orthogonal methods, of which 2 provided clinical diagnoses for patients’ phenotypes. They identified a paternally inherited pathogenic frameshift INDEL in *ATM* in an infant with T cell lymphopenia using trio exome sequencing. Pathogenic *ATM* variation causes ataxia-telangiectasia in an autosomal recessive manner but the patient’s exome data did not reveal a second trans variant in *ATM*. RNA sequencing of the patient’s fibroblasts identified reciprocal ATM-SLC35F2 and SLC35F2-ATM fusion transcripts suggesting chromosomal inversion that was later confirmed by targeted long-read sequencing of the putatively affected introns [136]. Recently, Cmero et al. showed how using RNA sequencing alone allowed discovery of an inter-chromosomal translocation in the *DMD* gene in a patient with muscular dystrophy [171]. Thus, integrated analysis of transcriptomic and genomic data should be considered to detect structural variants that may result in gene fusions.

### Long-read transcriptomics

Short-read RNA sequencing is a well-established and superior technique for gene expression quantification compared to microarray. However, the fragmented, short-length reads makes computational reconstruction of transcripts challenging, especially for complex genes or gene families containing many similar isoforms [79, 81]. Long-read technology can determine the sequence of full-length RNA transcripts by sequencing the cDNA (Pacific Biosciences and ONT) or the native RNA (ONT). The longer reads can span the sequence of the entire transcript and thus determine the underlying exon combinations [79, 81]. Therefore long-read RNA sequencing can improve the analysis of alternate splicing, potentially leading to discovery of novel isoforms and novel gene fusions. Recent studies have identified many new relevant isoforms using long-read RNA sequencing in healthy [172–174] and disease states [175, 176]. Long-read RNA sequencing also allows identification of allele-specific expression through haplotype phasing [177, 178].

The high depth required for clinical long-read RNA sequencing currently makes it cost inefficient for regular genetic diagnoses. Like DNA sequencing, targeted long-read RNA sequencing is a good alternative to investigate disease-relevant genes. Dainis et al. [179] performed targeted long-read genome and transcriptome sequencing to interrogate a putative splice-site-altering mutation in *MYBPC3* gene in a hypertrophic cardiomyopathy (HCM) patient. Comparing long-read transcriptomics data for *MYBPC3* from this HCM patient to that in three additional HCM patients and six control hearts, they identified two isoforms that were exclusively seen only in the patient under question. This study exemplifies how LRS can easily characterize alternatively spliced isoforms and link the improperly spliced transcripts to variant-associated alleles.

To summarize, transcriptome-wide long-read sequencing allows detection of full-length transcripts, alternative spliced isoforms, gene fusions, transcript-based haplotype phasing, allele-specific expression, and base modifications in RNA. Although, to the best of our knowledge, long-read RNA sequencing is yet to solve an undiagnosed disease, the technology holds the promise for rare Mendelian disorders, especially when the only one heterozygous variant is identified in a recessive disease.

### Single-cell transcriptomics

Although bulk RNA sequencing has the potential to identify the molecular cause and disease mechanism in rare disorders, it only captures average expression signal in the sample, which may comprise different cell types. In comparison, single-cell RNA sequencing (scRNA-seq) measures expression of genes within each cell, allowing researchers to study the sample heterogeneity and cell-to-cell variation. This enables discovery of new and rare cell types, improving our understanding of the disease mechanism. Montoro et al. used scRNA-seq on mouse tracheal epithelium to study cellular heterogeneity and identified a new and extremely rare cell type—pulmonary ionocyte [180]. They showed that these ionocytes expressed *CFTR* gene at much higher levels than any other cell type in both mouse and human airway tissue. Mutations in *CFTR* have been extensively reported in cystic fibrosis disease, and for years, the gene was thought to be expressed at low levels in ciliated cells that are common and distributed throughout the airway.

However, like most new technologies, scRNA-seq is associated with technical challenges (low capture efficiency and extremely sparse data) and high cost as compared to bulk RNA-seq [181, 182]. scRNA-seq data is sparse, with many observed zeros, indicating that a given gene in a particular cell has no unique molecular identifiers or reads mapping to it. This could represent real biology (truly silent gene) or a technical artifact (gene is expressed but was not detected by the scRNA sequencing) [181, 183]. One alternative approach to scRNA-seq is to extrapolate cellular components of the sample from bulk RNA-seq using deconvolution methods. There are more than 50 deconvolution methods published to date, that can be broadly categorized as marker-based (uses marker gene list for deconvolution), reference-based (for the deconvolution process, it uses cell type specific gene expression profiles and list of differentially expressed genes across the cell types in the reference), and reference-free (uses reference profiles for cluster annotation after the deconvolution step) [184–187].

scRNA-seq has enabled discovery of many rare, novel cell types or sub-cell populations or markers in many different tissues—like blood [188], brain [189, 190], pancreas [191], and cancer [192, 193] to list a few, and with continued improvement in the single-cell sequencing technology and algorithms, we anticipate its application to be extended to rare disease research in future. Comprehensive characterization of transcriptome in each cell may allow discovery of new cellular and molecular components in rare disease patients’ tissues and can be instrumental in elucidating the disease mechanism.

## Complementary technologies

Integrating sequencing data with other technologies can also provide leads to discover the underlying mutation in undiagnosed diseases where sequencing is inconclusive [194, 195]. Here, we provide examples from metabolomics [12, 196], methyl profiling [197], proteomics [194], and immunology [198–200] that assisted in a patient’s genetic diagnosis. The choice of assay is often driven by the patient’s phenotype.

### Methylation profiling

Epigenetic modifications like DNA methylation and histone modification have shown to have important implications in rare diseases like Immunodeficiency Centromeric instability Facial syndrome 1, Rett syndrome, and Rubinstein-Taybi [201–203]. Methylation profiling should be considered when there is suspicion of a genomic imprinting disorder or a VUS in a known methylation gene. Genomic imprinting is a phenomenon where a subset of autosomal genes is preferentially expressed from only one of the two parental chromosomes. This results from parental-specific methylation of cytosine at CpG dinucleotides of genes during gametogenesis [204, 205]. DNA methylation defects can be divided into two groups — epi-variants [206] and epi-signatures [207, 208]. Epi-variants involve a change in DNA methylation pattern of a small number of CpGs at a specific region of the genome whereas epi-signatures are unique combinations of DNA methylation changes at multiple loci across the genome and are specific for different genetic syndromes.

Technologies like RRBS, WGBS, and long-read sequencing can be used to assess genome-wide DNA methylation. Aref-Eshghi et al. [197] developed a machine learning model using genome-wide DNA methylation data from blood to predict 14 different Mendelian syndromes with neurodevelopmental presentations and congenital anomalies (ND/CA) that are associated with epi-signature. By applying this model to a cohort of 965 ND/CA patients, who previously had unrevealing conventional genetic testing including CNV microarray or ES, they identified 15 cases with one of the 14 Mendelian syndromes. They also identified 12 patients with imprinting and trinucleotide repeat expansion disorder and 106 cases with rare epi-variants in this cohort. This work led to development of EpiSign, a clinical-grade genome-wide DNA methylation assay for patients with developmental delay or suspicion of imprinting, trinucleotide repeat expansion, or one of the 50 methylation-related disorders [209]. In a recent study, Sadikovic et al. evaluated the clinical utility of EpiSign in a cohort of 207 patients that was divided into two subgroups — a targeted cohort, which included patients with inconclusive VUS and a screening cohort that comprised of patients with clinical findings consistent with hereditary neurodevelopmental syndromes but no previous conclusive genetic findings [210]. EpiSign enabled diagnoses for 35.3% (48/136) of participants in the targeted cohort and 11.3% (8/71) of those in the screening cohort.

Clinical interpretation of rare epi-variants remains challenging, especially those in intragenic regions or in genes not yet associated with the patient’s phenotype. Another limitation of this approach is the lack of large-scale databases of epi-variants in Mendelian diseases and population epigenome data that can be used as a reference and to differentiate between tolerant versus pathogenic epi-variants. Moreover, some of the disorders may not exhibit epi-signatures or epi-variants in blood and may be tissue specific.

### Metabolomics

For many rare unexplained metabolic disorders in children, where the causative variant was identified by ES, functional metabolomic studies have helped to uncover the disease mechanism [211–213] and even led to better disease management or treatment in some cases [24]. Tarailo-Graovac et al. [24] used targeted metabolomics to confirm the causality of mutations detected by ES in several individuals among a cohort of 41 patients with intellectual development disorder and unexplained metabolic phenotype. Splinter et al. [25] demonstrated how findings from metabolomics in an undiagnosed patient with multi-system disorder prompted re-analysis of exome sequencing data, followed by RNA sequencing, and led to the diagnosis. They identified consistently high levels of urinary organic acids in the patient, suggesting a deficiency in 3-hydroxy-3-methylglutaryl coenzyme A lyase (encoded by *HMGL* gene). Re-evaluation of the ES data identified a deletion in exon 1 of *HMGL*. RNA sequencing the patient’s fibroblast revealed a 50% lower level of HMGL expression as compared with fibroblasts from eight unaffected individuals. Although metabolomics and lipidomics can potentially provide diagnostic leads, the metabolic changes in rare and undiagnosed diseases may be subtle or confounded by a patient’s special diet or medication, making the analysis challenging.

### Proteomics

Proteins are the final component of central dogma and the effector molecules of a cell. Proteomics has a lower throughput as compared to other ‘omes, yet it can reveal impairment in protein synthesis, stability, degradation, and signaling, which may result in a disease state. Two broad categories of methods commonly used to study proteome are mass spectrometry-based and antibody-based techniques. In 2019, Grabowski et al. [194] demonstrated that mass spectrometry-based proteome analysis guided targeted genetic diagnostics and uncovered the underlying genomic mutations in two patients, which were initially missed by ES due to sequencing limitations. They studied the proteome of three rare monogenic diseases of neutrophil granulocytes — severe congenital neutropenia (SCN), leukocyte adhesion deficiency (LAD), and chronic granulomatous disease (CGD). They interrogated 4154 proteins from 16 patients with one of the three monogenic diseases of neutrophil granulocytes and 68 healthy controls. ES was unable to provide molecular diagnoses for two patients in this cohort, one with CGD and another patient with congenital neutropenia associated with albinism. For both the cases, top 10 deregulated proteins from the proteome analysis provided hints for the causative mutations — *NCF1* for CGD case and *RAB27A* for the second case with congenital neutropenia and albinism. These were missed by ES analysis because sequencing the *NCF1* gene is challenging as it shares 99% homology with two pseudogenes while re-examination of second’s patients sequencing data showed that the last part of exon2 in *RAB27A* was not covered by sequencing reads.

Antibody-based cytometry techniques — flow and mass cytometry — allow to study cellular heterogeneity and phospho-signaling within each cell. Although cytometry has not yet led to diagnosis in rare disease patients, they can provide molecular clues and improve our understanding of the disease, especially for inborn errors of immunity [214, 215]. Kanolkar et al. [200] showed how findings from flow cytometry (reduced phosphorylation of STAT1 in B cells upon IFN-ɣ stimulation and attenuated STAT5 phosphorylation in T cells upon IL-2 stimulation) in a patient prompted genomic analysis of 132 immunologically relevant genes that revealed a compound heterozygote mutation in IFNGR1 in the proband.

Many of the aforementioned technologies complement each other, fill the missing gaps, and better inform about the molecular pathophysiology of the disease. Although there are several successful examples of the integration of ES/GS with either transcriptomics [134, 142], metabolomics [25, 213], or proteomics [216], a single framework integrating different omics is lacking. Existing tools [217–220] for combining and analyzing multiple omics data were designed for the standard case-control studies and are not suitable for outlier-based analysis. Taking a systems biology approach by integrating results from several omics may further improve the diagnostic yield and our understanding of the disease’s molecular mechanism.

## Functional studies

Unraveling the molecular mechanisms of a putative disease-causing gene can help strengthen the case for causality and may provide insights for developing therapeutics. This can be achieved by modeling patients’ disease-causing variant or strong candidate variants in vivo using model systems like fruit flies (*Drosophila melanogaster*), nematode worm (*Caenorhabditis elegans*), zebrafish (*Danio rerio*), mouse (*Mus musculus*)) [221–224], or in vitro (disease-relevant mouse or human cell lines, primary cells or induced pluripotent stem cell models, iPSCs). Such models can be a fast and cost-effective way to mirror complex rare genetic disorders. The choice of the model system depends on the cost, time, and the ability to model and assess the patient’s phenotype in the animal [223].

Several groups have used organisms like Drosophila, *C. elegans*, and zebrafish to model patient’s mutations to (i) validate novel disease-gene associations [225], (ii) provide functional data [226], (iii) generate new biological insights [227], and (iv) even identify potential therapeutic targets [228, 229]. Splinter et al. [25] demonstrated that modeling candidate variants in Drosophila and zebrafish played an important role in the diagnoses of eight patients in a cohort of 382. Functional studies in Drosophila confirmed causation of a de novo variant in *NR5A1* gene in a patient with a 46,XX genotype and male sex characteristics [230], which later led to characterization of a new syndrome. Similarly, Kanca et al. [231] performed functional studies in Drosophila to establish that de novo variants in *WDR37* gene cause a novel syndromic neurological disorder and Ferreira et al. [232] used zebrafish to model variants causing Saul-Wilson syndrome.

Another useful resource to recapitulate the unique aspects of patients’ disease pathology is their own cells, which can be grown into fibroblasts or induced pluripotent stem cells (iPSCs) [233]. iPSCs can be potentially differentiated into virtually any cell type with the appropriate environmental stimuli. iPSCs are especially valuable for rare disorders that affect inaccessible tissues such as neurons [234] and cardiomyocytes [235]. Modeling of disease-relevant cell types has allowed better understanding of disease pathogenesis in many rare diseases [233] like those involving neurons (ALS [236], Friedreich’s ataxia [237], ataxia-telangiectasia [238]), cardiomyocytes (long QT syndrome [239], Fabry disease [240], Jervell and Lange-Nielsen syndrome [241, 242]), blood (Fanconi anemia [243], Glanzmann thrombasthenia [244]), connective tissue (Fibrodysplasia ossificans progressiva [245]), and eye (Retinitis pigmentosa [246, 247]). Yamashita et al. [248] used iPSCs to model monogenic skeletal diseases like Thanatophoric Dysplasia type 1 (TD1) and achondroplasia (ACH) and to identify clinically effective treatment for these diseases. The authors converted fibroblasts from TD1 and ACH patients into iPSCs and demonstrated that the chondrogenic differentiation of TD1 iPSCs and ACH iPSCs resulted in the formation of degraded cartilage. Next, they showed that statins, a class of drugs already approved for lowering lipids, could correct the degraded cartilage in both chondrogenically differentiated TD1 and ACH iPSCs. These results were then reproduced in mice, suggesting that statins might be an effective drug for patients with TD1 and ACH.

ES and GS yield numerous VUS, non-coding variants within functional regulatory elements and variants that disrupt splicing. Existing computational prediction algorithms have had limited success in prioritizing them. Functional screening assays are a powerful platform to assess the impact of variants in thousands of genes in a single experiment. Such screening approaches include germline mutagenesis, CRISPR/CAS9, plasmid-based reporter assays, RNA interference, chemical screens [249], and multiplexed assays of variant effect [250, 251]. Advancements in CRISPR/Cas9 technology make it a robust tool to profile cellular phenotypes resulting from each of the thousand genetic perturbations in a high-throughput manner [252]. The underlying principle behind CRISPR screens [253, 254] is to introduce thousands of variants in a large cell population but only one gene is perturbed per cell. This results in a population of cells with a different gene disrupted in each cell. Then, sequencing is performed on the mixed population of cells to identify genetic sequences necessary for the cell’s survival or a specific cellular phenotype of interest. CRISPR screens target multiple sites per gene and thus introduce random variants in the gene of interest that may not represent the exact mutation observed in the patient. However, this approach informs whether a particular genomic region may have a functional role in the disease and can narrow down to a few promising candidates for follow-up studies. CRISPR screens can be implemented to study the effect of knockout (CRISPRko), inhibition (CRISPRi), or activation (CRISPRa) of many protein-coding genes [253, 255] or non-coding regulatory elements [256, 257].

CRISPR screens have recently been used to link new genes to rare diseases [258], to understand the molecular mechanism through which different variants in a gene contribute to a disease [259] and to explore potential therapeutic targets [260]. Rao et al. [259] used a pooled CRISPR screen in human hematopoietic stem and progenitor cells (HSPCs) to study mutations in *ELANE* which is known to cause severe congenital neutropenia (SCN), a rare genetic disorder characterized by low circulating neutrophils caused by impaired neutrophil maturation. Missense and frameshift mutations in *ELANE* account for 50% of SCN cases. The authors performed a dense mutagenesis CRISPR screen in primary human HSPCs to identify *ELANE* variants associated with neutrophil maturation defects. Although CRISPR screens hold great promise for prioritization of variants identified by ES and GS, there are some practical limitations including the need to study a large number of cells (10^8^) and the fact that current CRISPR screens may not model the specific prioritized variants from the patient.

Therefore, functional studies including model organisms, fibroblasts, iPSCs, and CRISPR/CAS9 screens can play a vital role in diagnosis, understanding the molecular mechanism of rare and undiagnosed diseases and exploring potential therapeutic strategies. However, each model system has its own limitations. This process is time consuming, and moreover, none of the model systems can completely replicate human disease.

## Case matching

A major challenge faced by rare disease researchers is the lack of phenotypically similar patients to establish the molecular cause of the disease and to conduct statistical analysis. Several algorithms and platforms [261–264] have been developed to discover cases with common phenotypes and disrupted genes. However, there was a lack of a federated network that would facilitate interaction between various rare disease databases in a streamlined and continuous manner. To address this, Matchmaker Exchange (MME) was launched in 2015 [265] to identify unrelated cases with a potentially pathogenic variant in the same candidate gene and overlapping phenotype. It performs genomic matching across several databases (like DECIPHER [266], GeneMatcher [267], PhenomeCentral [264]) in a scalable, secure, and automated fashion through a standardized application programming interface (API). As of October 2021, MME contains information from more than 150,000 cases from 88 countries [268]. It has facilitated identification of cases with similar phenotypic and genotypic profiles for many rare diseases including 25 novel gene-disease associations and phenotype expansions. MME also allows queries against published animal models that match a patient’s phenotype, connecting the clinician with model organism researcher.

Some rare mutations can cause dysmorphic and unique facial features. Integrating the patients’ variant and phenotypic data with their facial features (images) can significantly narrow down the search for potential rare syndromes. Using 17,000 images representing more than 200 syndromes, Gurovich et al. [269] developed a deep neural network to classify distinctive facial features in photos of patients with congenital and neurodevelopmental disorders. Machine learning is also being applied to large electronic health record (EHR) databases to identify rare as well as common disease patients [270–272]. This can help to find patients who have similar disease trajectories (like symptoms, age, medications, labs, or procedures) but may not have a definite diagnosis.

## Automated re-analysis

It is important to periodically re-analyze sequencing data with latest analytical pipelines, variant frequency databases, literature [273–275], and updates in patient’s phenotype that can potentially identify recent associations between the causative gene and patient’s symptoms. Several groups have demonstrated that re-evaluation of genomic data can increase the diagnostic yield: by 5–26% in case of ES [276–279] and by 4–11% for GS [280, 281] re-analysis. Use of standard ontologies like Human Phenotype Ontology (HPO) [282] can help to prioritize candidate genes that have been previously linked to the patient’s phenotype. Tools like exomiser [283], amelie [284], and Xrare [285] search for the gene-phenotype associations in human diseases (documented in databases like OMIM [286], Orphanet [287], or PubMed) and also in other models like mouse, zebrafish, and protein-protein interaction networks. Using ES from 134 diagnosed rare retinal diseases, Cipriani et al. [288] demonstrated that exomiser tool ranked the causative variant as top hit in 74% of the dataset and among top 5 in 94%. Deeply phenotyping undiagnosed patients and identifying the most relevant symptoms is critical. However, these patients have an extensive, complex medical history, and their symptoms are documented in long clinical records. Tools like ClinPhen [289] and CLiX [290, 291] can extract relevant phenotypes from clinical notes or EHR data and convert them to HPO terms, thus enabling development of an automated pipeline for phenotype-based prioritization of variants.

Along with improving the diagnostic yield for rare disease patients, clinicians and rare disease researchers would like to reduce the diagnosis time frame for all patients. It is obvious that delay in accurate diagnosis leads to inappropriate disease management and sometimes even unnecessary treatments that can have severe side effects. To scale genomic analysis and implement it at a clinical level in a secure fashion, many groups are leveraging the power of cloud computing (like Amazon Web Services [https://aws.amazon.com/], Google Cloud Platform [https://cloud.google.com/]), and even new hardware like DRAGEN (Dynamic Read Analysis for Genomics) has been designed for faster turnaround of results. DRAGEN implements FPGA (Field Programmable Gate Array) technology, an alternative to conventional CPU-based systems to expedite the execution of genome pipelines. Recently, some groups have demonstrated record-breaking fast implementation of GS using optimized SR/LR sequencing, DRAGEN/multiple cloud computing machines, and semi-automated downstream analysis to diagnose children with suspected Mendelian disorder, who were critically ill and admitted to ICU (fastest, 7 h, 18 min) [292, 293].

## Challenges

Accurately diagnosing rare disease patients involves challenges at technical, financial, and policy levels. Some of the technical obstacles include interpretation of non-coding variants and VUS. This requires use of advanced technologies (like LRS, RNA-seq, epigenomics) and algorithms (like SplicsAI, genomiser) to decipher the role of non-coding regions in healthy and disease conditions. Periodic and automated re-analysis of genomic data can help to resolve some of the VUS and intronic variants as new disease-gene discoveries are being made at an accelerated pace. There is also a need for specialized methods to analyze and integrate multiomics data for rare diseases.

In 2016, Kessler et al. [294] demonstrated ancestry specific bias in genomic datasets and its impact on diagnostic accuracy and cost. Human variant databases like 1000 Genomes [295], ESP [296, 297], and Exac [298] catalogs the frequency of variants from large populations. Mutations absent or present at very low frequency in these databases are prioritized as potential causes of rare Mendelian diseases, based on the assumption that variants common in the general population are unlikely to cause a rare or undiagnosed disease. However, these databases are skewed towards European ancestry populations [299–301] which makes interpretation of ES/GS from an individual with non-European inheritance more difficult, expensive, and time consuming. Recent efforts like gnomAD [302], GenomeAsia 100 K [303], and All of Us [304] have been initiated to sequence more diverse and underrepresented populations. Thus, it is critical to query the allele frequencies of non-European patients from their respective ancestry and for the scientific community to fill the ethnicity gaps in the current genomic databases. A complementary approach is the use of the aforementioned pan-genome reference. Encoding the genetic diversity in the reference genome would benefit genomic analysis of non-European ethnicities by reducing the reference bias during alignment and thereby resulting in a more accurate variant calling [119, 124, 125].

One of the biggest challenges in the road to diagnosing rare disease patients is the cost. The technologies mentioned in this manuscript are often not available clinically or covered by patients’ medical insurance [305] and are provided by few research programs in developed nations. Dimmock et al. [306] reported that rapid GS improved the disease management in 58 children in a cohort of 184 critically ill infants, who were admitted to ICU and reduced the hospital costs in 31 cases, by $12,000–$15,700 per child. Splinter et al. [25] compared the health care cost before and during the diagnosis evaluation period and found the latter to be only 6–7% of the total cost. Recently, Tisdale et al. [307] performed a pilot study on 14 rare diseases within four different healthcare system databases to estimate direct medical costs. They found that per patient direct medical costs of rare diseases are about 3–5 times higher than age matched controls, highlighting the urgent need for early and accurate diagnosis for rare disease patients that may reduce the costs associated with misdiagnosis or missed opportunities for intervention at an appropriate time. More of such cost-effectiveness analyses are required to justify the cost of whole genome SRS or LRS to be covered by insurance and to bring a change in the policy. Also, there is a need for continuous funding to the existing research programs dedicated for diagnosing rare and yet-to-be-discovered diseases. With continued advancement in the technologies, we anticipate a decline in their cost will make them more affordable. Meanwhile, targeted sequencing and latest computational algorithms should be considered to address the challenges of detection and interpretation of genomic variants, along with machine learning approaches to identify similar patients.

## Conclusions

In the past two decades, gene panels, microarrays, and ES have identified the underlying causal mutations for many rare disease patients; however, still a significant proportion of them remain undiagnosed. In this review, we summarize different approaches that can further improve the diagnostic yield and elucidate the molecular mechanism of the disease. We share examples where these technologies played a significant role in deciphering the causative mutation in undiagnosed patients.

These approaches include complementing short-read genome sequencing with RNA sequencing, metabolomics, proteomics, and methyl profiling. For patients with unrevealing short-read GS, long-read technology is a promising alternative. It is also important to functionally validate the candidate or causative variants identified through genomics using in vitro and in vivo model systems to improve our understanding of molecular mechanisms and to allow better disease management, even opening avenues towards therapeutics.

It is also critical to periodically implement fast, automated computational pipelines to identify new gene-disease associations or to find similar patients across the globe. Lately, the medical and genomics community has recognized and acknowledged the ancestry specific bias in the genomic datasets and in the haploid linear reference genome. Inclusion of diverse ethnicities in frequency databases and use of a pan-genome reference will help to improve the diagnostic accuracy for underrepresented populations.

A major bottleneck in the diagnosis of rare patients is the cost involved in the investigation: most of the assays mentioned in this review are research based and not yet available through health care systems. We anticipate that continuous improvements in accuracy and affordability of the high-throughput technologies will enable us to fill the diagnostic gap for undiagnosed patients, often with actionable findings. We envision that successful implementation of complementary multidisciplinary studies will lead to a paradigm shift in how undiagnosed patients are diagnosed and treated.

## Data Availability

Not applicable.

## Abbreviations

ES: Exome sequencing
GS: Genome sequencing
SVs: Structural variants
bp: Base pairs
UTRs: Untranslated regions
IRD: Inherited retinal disease
STRs: Short tandem repeats
PCR: Polymerase chain reaction
SRS: Short-read sequencing
LRS: Long-read sequencing
SMRT: Single-molecule real-time
ONT: Oxford Nanopore Technologies
HiFi: High-fidelity
WGBS: Whole genome bisulfite sequencing
RRBS: Reduced representation bisulfite sequencing
ASE: Allele-specific expression
VUS: Variant of unknown significance
TSVs: Transcriptomic structural variants
TD1: Thanatophoric Dysplasia type 1
ACH: Achondroplasia
EHR: Electronic health record
HPO: Human Phenotype Ontology
DRAGEN: Dynamic Read Analysis for Genomics
FPGA: Field Programmable Gate Array
iPSCs: Induced pluripotent stem cells
SNVs: Single-nucleotide variants
INDELs: Insertion and deletion
TELL-seq: Transposase Enzyme Linked Long-read Sequencing
stLFR: Single-tube long fragment read
